# Investigation of potential migratables from paper and board food contact materials

**DOI:** 10.3389/fchem.2023.1322811

**Published:** 2023-11-30

**Authors:** Mélanie Di Mario, Gregory Bauwens, Florian Peltier, Séverine Goscinny, Jean-François Focant, Giorgia Purcaro, Els Van Hoeck

**Affiliations:** ^1^ Organic Contaminants and Additives Service, Sciensano, Brussels, Belgium; ^2^ Analytical Chemistry Lab at the AgroBioChem Department, Gembloux Agro-Bio Tech, University of Liège, Gembloux, Belgium; ^3^ Organic and Biological Analytical Chemistry Group, MolSys Research Unit, University of Liège, Liege, Belgium

**Keywords:** paper and board FCM, plasticizers, photoinitiators, bisphenols, primary aromatic amines, mineral oil, liquid chromatography, gas chromatography

## Abstract

Since the ban on single-use plastic articles in Europe, the food contact material (FCM) industry has been forced to move to more sustainable alternatives. Paper and board FCM are convenient alternatives but must be safe for consumers. This study aims to investigate potential migrations of various substances (e.g., plasticizers, photoinitiators, primary aromatic amines, mineral oil, and bisphenols) from straws and takeaway articles made of paper and board. Twenty straws and fifty-eight takeaway articles were carefully selected and investigated using liquid and gas chromatography coupled with tandem mass spectrometry or flame ionization detector. Fourteen substances of all the targeted categories were found in takeaway articles, including seven plasticizers, two photoinitiators, one primary aromatic amine, two bisphenols, and the saturated and aromatic fraction of mineral oil (MOSH and MOAH, respectively). In straws, fewer substances were detected, i.e., six substances, including three plasticizers, one photoinitiator, MOSH, and MOAH. At least one of the target substances was detected in 88% of the samples, demonstrating the importance of further evaluation of these materials. Finally, the associated risks were assessed, highlighting the potential risks for several types of articles regarding bisphenol A, one primary aromatic amine (3.3-DMB), and MOSH and MOAH.

## 1 Introduction

Food contact materials (FCM) are often fossil-based, i.e., plastic (Elias and Brennan, 2008). In 2021, the production of plastic was estimated at 390 million tons worldwide (Plastics Europe, 2019). However, there has been increasing concern about the behavior and fate of these materials when discarded into the environment (Geueke et al., 2018). Consumer awareness is rising, and plastic FCM is pinpointed as a major environmental problem. Consequently, the FCM industry is under considerable public pressure to replace fossil-based plastics. This concern is shared at the European level, and recently, the European Parliament and the Council imposed the reduction of the impact of certain plastic products on the environment (EU Publications, 2019). Since 3 July 2021, certain single-use plastic articles have been banned in EU Member States, and all plastic packaging should be reusable or easily recyclable by 2030 in accordance with the EU strategy for Plastics in the Circular Economy (European commission, 2018). At the Belgian level, the Federation of Belgian Food Industry (FEVIA) states that all food packaging must be reusable, recyclable, or compostable by 2025 (Fevia, 2018). One way to tackle the waste problem is to introduce environmentally friendly FCM, which must also be safe for consumers. Various alternatives have been developed, focusing on bioplastics or recycled materials. Alternatively, the field of application of other materials has been extended. For example, straws made of single-use plastic have been replaced by paper straws. Paper and board FCM offer a significant advantage, i.e., versatility. Indeed, depending on the coating or treatment of the material, they can either be used for liquid, frozen, fresh, or dry foods. In addition, paper and board is the main material used in fast-food restaurants. From an environmental point of view, paper and board FCM are made from natural and renewable raw materials. Consequently, these materials could be recycled, biodegraded, or incinerated if no non-biodegradable coating is present.

According to the European Association representing the paper industry (Cepi), paper and board production in Europe increased by 6.1% in 2021 compared to 2020, reaching 90.5 million tonnes (Cepi, 2021). Although paper and board FCM are gaining popularity, the legislative framework encounters some limitations. All FCMs are regulated at the European level under the Framework Regulation (EU) No 1935/2004 and Regulation (EC) No 2023/2006. However, specific EU Regulation only exists for plastic FCM (Commission Regulation, 2011). For paper and board, specific national legislations exist in several countries like Switzerland (Das Eidgenössische Departement des Innern, 2022), Italy (Il ministro della salute, 2007), Germany (Bundesinstitut für Risikobewertung, 2015), Netherlands (van Volksgezondheid and en Sport, 2014) and France (DG CCRF, 2019). The Council of Europe also published a technical guide on paper and board as FCM, providing advice on compliance testing and supporting documentation (EDQM, 2021). These different legislations and recommendations have in common that the safety of the consumer should be guaranteed. Therefore, it is crucial to evaluate the migration of potentially harmful substances. These substances can be intentionally added, e.g., additives, synthetic fibers, adsorbents, treatment agents, and colorants, or be present unintentionally, like degradation products or substances originating from the recycling process. Furthermore, paper and board FCMs are often coated, glued, printed, composed of several layers, or combined with other materials (EDQM, 2021). Identifying the origin of certain substances can be quite challenging. Certain phthalates, for example, are ubiquitous in the environment, making it difficult to determine where contamination originated. Phthalates may come from the production or recycling process, while others may be the result of environmental contamination. Similarly, bisphenols seem to be more prevalent in recycled fibres than in virgin fibres, suggesting that part of the contamination may originate from the recycling process. Mineral oil hydrocarbons are since few years of great concern. They can either contaminate food due to the use of lubricants for machinery, through environmental contamination, migration from food contact materials etc (Schrenk et al., 2023).

Over the past 10 years, paper and board FCM have been studied extensively. Many contaminants, such as phthalates (Fierens et al., 2012; Pivnenko et al., 2016; Vápenka et al., 2016; Vavrouš et al., 2016; Van Den Houwe et al., 2017), printing inks (Bradley et al., 2013; Van Den Houwe et al., 2016; Vápenka et al., 2016; Van Den Houwe et al., 2017), bisphenols (Pérez-Palacios et al., 2012; Suciu et al., 2013; Jurek and Leitner, 2017) as well as mineral oil hydrocarbons (MOH) (Biedermann and Grob, 2010; Dima et al., 2011; Vollmer et al., 2011; Zurfluh et al., 2013; Fengler et al., 2015; Pivnenko et al., 2016), were often reported in the literature as contaminants. Perez-Palacios et al. showed the presence of bisphenol A (BPA) in recycled paper and board hamburger boxes, milk bricks, pizza boxes, and packaging (Pérez-Palacios et al., 2012). Suciu et al. investigated the presence of BPA and di-2-ethyhexyl phthalate (DEHP) in cereals, rice, polenta eggs, salt, sugar, vegetables, and pizza boxes. Bisphenol A was present in all samples, and DEHP was quantified in 70.5% of the samples (Suciu et al., 2013). Jurek et al. (Jurek and Leitner, 2017) and Vavrous et al. (Vavrouš et al., 2016) detected multiple bisphenols (BPA, BPE, BPF, and BPS) in recycled and virgin paper and board FCM (paper bags for bakery products, pizza boxes, cheese paper, etc.). The content of MOH in paper and board has already been well investigated for dry food sold on the market (Vollmer et al., 2011; Grob, 2018). However, limited data exist on takeaway articles or straws. (Fengler et al. (2015). conducted a study on the migration of MOH from fast food packaging, and Mineral Oil Saturated Hydrocarbons (MOSH) and Mineral Oil Aromatic Hydrocarbons (MOAH) were found in all the samples (pizza boxes, hamburger boxes, fries trays and a wrap). Conchione et al. (2020) focused on pizza boxes from pizza restaurants and takeaway pizzas. MOAH was found in 89.5% of the samples, while MOSH was detected in all samples. Recycled fibers (forbidden according to Italian legislation), printing inks and refined paraffin were identified as sources of contamination of pizza boxes in this study.

Exposure to these substances can present a considerable risk to human health due to their endocrine-disrupting, carcinogenic, mutagenic, reprotoxic, or bioaccumulative properties.

Furthermore, nowadays, consumers have changed their habits, especially since the COVID-19 crisis. Takeaway has exploded (Kochańska et al., 2021). Additionally, consumers’ awareness of environmental issues leads them to use more environmental-friendly materials. Therefore, assessing the risk of new articles frequently used in daily life, like straws and takeaway articles, is crucial.

Accordingly, this study aims to investigate and assess the risk of potential migrations of bisphenols, plasticizers, primary aromatic amines, photoinitiators, and mineral oil from straws and takeaway articles made of paper and board.

## 2 Materials and methods

### 2.1 Sampling

This study focused on straws and takeaway FCM. Therefore, the sample selection was based on (i) a web-based market study of fifty-two websites in Belgium (Ciano et al., 2023); (ii) articles purchased in Belgian supermarkets (i.e., packaging with a straw, like milk boxes); and (iii) articles available in fast food restaurants. A total of 20 straws and 58 takeaway articles were selected.

### 2.2 Solvents, reagents, and standard solutions

#### 2.2.1 Primary aromatic amines, plasticizers, photoinitiators, and bisphenols

All the analytical standards were purchased from Sigma Aldrich (Saint-Louis, United States). The targeted substances as well as their purity are listed in Supplementary Table S2. Stock solutions, at a concentration of 1 mg mL^-1^, were prepared in acetonitrile for plasticizers, while methanol was used for photoinitiators (PIs), bisphenols (BP), and primary aromatic amines (PAAs). Next, working solutions were prepared by appropriate dilutions of the stock solutions with acetonitrile for phthalates, bisphenols, and photoinitiators and in methanol for primary aromatic amines. All solutions were stored at −20°C and kept for 3 months. Except for PAAs, for which the working solutions were prepared daily.

Methanol HPLC grade 99.9% (MeOH), acetonitrile HPLC grade 99.9% (ACN), Ethanol 96% (EtOH), hexane 99.0%, and formic acid 99.0% were purchased from Biosolve (Valkenswaard, The Nederlands). Water was purified using a Millipore Milli-Q IQ 7000 system (Merck, Overijse, Belgium).

The results were quantified using a calibration curve prepared in 95% EtOH for plasticizers and Milli-Q water for primary aromatic amines, bisphenols, and photoinitiators. The following working range was applied: plasticizers (from 10 to 100 ng mL^-1^), bisphenols (from 10 to 1,000 ng mL^-1^), primary aromatic amines (from 0.5 to 30 ng mL^-1^), and photoinitiators (from 5 to 150 ng mL^-1^). The LOQs are presented in Supplementary Table S1.

The mass spectrometer parameters of the targeted substances can be found in Supplementary Table S2.

#### 2.2.2 Mineral oil

Solvents were purchased from Merck Millipore (Hoeilaart, Belgium). The MOSH and MOAH internal standards (IS), containing 5-α-cholestane (Cho, 0.6 mg mL^-1^), n-C11 (0.3 mg mL^-1^), n-C13 (0.15 mg mL^-1^), cyclohexyl cyclohexane (CyCy, 0.3 mg mL^-1^), n-pentyl benzene (5B, 0.30 mg mL^-1^), 1-methyl naphthalene (1-MN, 0.30 mg mL^-1^), 2-methylnaphthalene (2-MN, 0.30 mg mL^-1^), tri-tert-butyl benzene (TBB, 0.3 mg mL^-1^) and perylene (Per, 0.6 mg mL^-1^) in toluene, and the MOSH/MOAH retention time standard, containing a standard mixture of n-alkanes (C10, C11, C13, C16, C20, C24, C25, C25, C35, C40, and C50, 50 mg L^-1^ each, were provided by Restek (Neukirchen-Vlun, Germany). The glassware was carefully washed and rinsed before use with distilled solvents (acetone and n-hexane). All the analytical standards were purchased from Sigma Aldrich (Saint-Louis, United States).

### 2.3 Sample preparation and analysis

#### 2.3.1 Primary aromatic amines, plasticizers, photoinitiators, and bisphenols

The experiments were performed according to the guideline on testing conditions for kitchenware articles in contact with foodstuffs, developed by the European Reference Laboratory (EURL) for Food Contact Materials (Beldi et al., 2023). More specifically, investigating the potential migratables was based on the CEN standards EN645 (Swedish standard SS-EN 645, 1994) and EN15519 (European Standard, 2008). The intact article was extracted when article filling (e.g., cups, bowls) or immersion (e.g., straws) was possible. If not possible, a piece of 1 dm^2^ was cut and immersed in the extraction solvent. Two procedures were applied to all the samples. One sample was extracted for 24 h at 23°C with distilled water, while a duplicate sample was extracted for 2 h at 20°C with 95% ethanol. Except for the cups, the ethanol experiment was performed for 24 h. Afterwards, liquid chromatography coupled to a tandem mass spectrometer (LC-MS/MS) was used to analyze primary aromatic amines, bisphenols, and photoinitiators in the water extract, while gas chromatography coupled to a tandem mass spectrometer (GC-MS/MS) was used for the analysis of plasticizers in the ethanol extract. The chromatography and mass spectrometry details are available in the Supplementary Tables S2–S5.

#### 2.3.2 Mineral oil

The samples were analyzed according to the procedure, developed by the BfR (Bundesinstitut für Risikobewertung, 2012). Briefly, 2 g of each FCM were chopped with a length of around 0.5 mm (the representative sample should not include glued parts (e.g., hotmelts)). The 2 g of sample were mixed with 20 µL of IS and 10 mL of a mix of hexane and ethanol (hexane/EtOH 1/1 v/v) using a magnetic stirring bar for 5 min. Next, the vessels were kept for 2 h at room temperature before shaking again for 5 min. The extraction time was extended to 24 h for samples containing plastic (Vollmer et al., 2011). Five mL of the extract were recovered and mixed with 10 mL of H_2_O to remove the EtOH. 1.5 mL of the organic phase was put into a vial for injection. 100 μL was injected into the instrument.

When needed, the ALOX procedure was applied (Online Browsing Platform, 2023). Briefly, a glass column was filled with 10 g of aluminium oxide, 3 g of silica gel, and 1 g of sodium sulfate. The column was pre-cleaned with 20 mL of hexane, and 0.5 µL of the sample was loaded. The collection began just before the loading of the sample, and the elution was performed with 25 mL of hexane. After that, the eluate was evaporated under a vacuum at 35°C after adding a keeper (2 drops of bis(2-ethylhexyl)maleate. The residue was dissolved in 500 µL of hexane, and 100 µL was injected. Integration was performed using CyCy and 1 MN for MOSH and MOAH, respectively.

The LC-GC analyses were carried out in a fully integrated platform, as reported in Bauwens et al. (Bauwens et al., 2023a; Bauwens et al., 2023b) consisting of an Agilent 1260 Infinity II LC equipped with an isocratic pump G7110B and a Variable Wavelength Detector acquiring at 230 nm (Agilent Technologies, Waldbronn, Germany). The pump was modified by Axel-Semrau to ensure the minimization of dead volumes. A 250 mm × 2.1 mm i.d. × 5 μm *dp* Allure silica column (Restek, United States) was used for the LC separation. The GC column set consisted of an MXT-1 (non-polar) 15 m × 0.25 mm i.d. × 0.1 μm *df* (Restek) connected to a Select PAH column (mid-polar) of 0.9 m × 0.15 mm i.d. × 0.1 μm *df* (Agilent Technologies). This column configuration allowed to quickly move from LC-GC to LC-GC×GC by switching on and off the modulator, assuring no shift of the MOAH fraction compared to the MOSH one. All chromatographic conditions were as reported in Bauwens et al. (Bauwens et al., 2023b). The analyses were all conducted in LC-GC-FID.

### 2.4 Expression of the results

According to Article 17 of Regulation (EU) 10/2011, the analysis results should be expressed in mg kg^-1^ food, applying the surface-to-volume ratio in actual or foreseen use. For articles containing or immersed in less than 500 mL or more than 10 L, the results are expressed in mg kg^-1^ food, applying a surface-to-volume ratio of 6 dm^2^ per kg of food.

### 2.5 Risk assessment

The risk assessment was performed according to the RACE tool (Rapid Assessment of Contaminant Exposure tool) developed by EFSA (Fürst et al., 2019). First, toxicological information (e.g., Health Based Guidance Values) of the migrants was collected from different sources. If available, the tolerable daily intake (TDI) of each migrant was collected from EFSA opinions (European Food Safety Authority (EFSA), 2009; EFSA Panel on Food Contact Materials, Enzymes and Processing Aids (CEP), 2019; Lambr et al., 2023). If a Health Based Guidance Value was not available, a reference point (e.g., No Observed Adverse Effect Level (NOAEL)) was searched. In that case, a higher level of uncertainty is assumed. In cases where no or very limited toxicological information is available, the threshold of toxicological concern (TTC) approach was used. For substances that can potentially be DNA-reactive mutagens and/or carcinogens, the TTC value is set at 0.0025 μg kg^-1^ body weight (bw) per day and was set based on the carcinogenic potency database (More et al., 2019). All other substances, except organophosphates or carbamates, are grouped according to the Cramer classifications. The TTC values for Cramer Classes I, II and III are 30 μg kg^-1^ bw per day, 9 μg kg^-1^ bw per day and 1.5 μg kg^-1^ bw per day, respectively (More et al., 2019).

An overview of the toxicological information used for the risk assessment is given in Table 1.

**TABLE 1:** Toxicological information of the substances.

Substances	Toxicological information	TTC threshold	References
BPA	TDI: 0.0002 μg kg^−1^ bw day		Lambr et al. (2023)
BPS	NOAEL: 20 mg kg^−1^ bw day		More et al. (2022)
3,3-DMB	No available toxicological information	0.0025 μg kg^−1^ bw day	*—*
DBP	TDI: 10 μg kg^−1^ bw day		EFSA Panel on Food Contact Materials, Enzymes and Processing Aids (CEP) (2019)
DiBP	TDI: 10 μg kg^−1^ bw day		^a^see text below
BBP	TDI: 500 μg kg^−1^ bw day		EFSA Panel on Food Contact Materials, Enzymes and Processing Aids (CEP) (2019)
DEHP	TDI: 50 μg kg^−1^ bw day		EFSA Panel on Food Contact Materials, Enzymes and Processing Aids (CEP) (2019)
DINP	TDI: 150 μg kg^−1^ bw day		EFSA Panel on Food Contact Materials, Enzymes and Processing Aids (CEP) (2019)
DIDP	TDI: 150 μg kg^−1^ bw day		EFSA Panel on Food Contact Materials, Enzymes and Processing Aids (CEP) (2019)
DINCH	TDI: 1,000 μg kg^−1^ bw day		Lambr et al. (2023)
MOSH	NOAEL: 236 mg kg^−1^ bw day		Schrenk et al. (2023)
MOAH	BMDL10: 0.49 mg kg^−1^ bw day		Schrenk et al. (2023)
Benzophenone	TDI: 30 μg kg^−1^ bw day		European Food Safety Authority (EFSA) (2009)
HCPK	No available toxicological information	30 μg kg^−1^ bw day	*—*

^a^ For DiBP, no TDI was set by EFSA, but the CEP Panel concluded that DIBP substantially added to the overall exposure and risk of consumers to phthalates, noting the similar (i) potency concerning reproductive effects and (ii) intake estimates compared to DBP (as outlined in the ECHA RAC assessment of 2017) (Fürst et al., 2019). Therefore, the TDI, of DBP was also applied to DiBP.

Next, according to the EFSA guidance, three age categories were targeted: Children (3–10 years old, 23 kg), teenagers (14–18 years old, 61 kg) and adults (18–64 years old, 70 kg) (EFSA Scientific Committee, 2012). In order to perform a proper risk assessment, hypotheses on the consumption of food intended to be in contact with the targeted articles were determined, thereby assuming complete migration as determined in Sections 3, 4 at every exposure occasion. An overview of the hypotheses is given in Supplementary Table S6.

Finally, all information was combined to perform the risk assessment of the migrants. The calculated exposure was compared to this threshold for the substances with a TDI. When the exposure exceeds the threshold, there is a potential risk for the consumer. When a TDI was not available, but another reference point was used for the risk assessment, the reference point was divided by the calculated exposure. The EFSA Working Group considers that, in general, a margin of at least 300 would be adequate to ensure a low concern for public health (Bauwens et al., 2023a). However, other values can be applied when deemed necessary.

## 3 Results and discussion

Since the ban on single-use plastic articles in Europe, FCM industries have been forced to move to more sustainable alternatives. Paper and board FCM are convenient alternatives but must be safe for consumers. One of the FCM categories with the largest shift from single-use plastics towards paper and board is the category of takeaway articles and straws. Therefore, a sampling of these types of FCMs was performed. Overall, 20 straws and 58 takeaway FCM were selected, covering the majority of articles available on the market. The different groups associated with the number of articles purchased per category are presented in Table 2.

**TABLE 2:** Overview of the selected samples.

Category	Examples	Number
Straws	Cocktail straws, juice straws, soda straws, etc.	20
Boxes	Pizza, noodles, hamburger boxes	16
Trays/bags	Fries, snack trays/bags	13
Wraps/bags	Hamburgers, tacos, sandwiches, wraps	7
Cups	Soda, coffee cups	13
Bowls	Soup, ice cream, salad bowls, etc.	7
Utensils	Spoon	2

More detailed information about the samples, based on the labelling (*e.g.*, type of FCM, presence of recycled fibers, coating, colour, etc.), is described in Supplementary Table S7. Next, the samples were extracted and analyzed for various substances. Previous studies on paper and board often indicated the presence of bisphenols, phthalates, photoinitiators and mineral oil (Biedermann and Grob, 2010; Vollmer et al., 2011; Fierens et al., 2012; Pérez-Palacios et al., 2012; Bradley et al., 2013; Suciu et al., 2013; Zurfluh et al., 2013; Pivnenko et al., 2016; Van Den Houwe et al., 2016; Vápenka et al., 2016; Vavrouš et al., 2016; Jurek and Leitner, 2017; Van Den Houwe et al., 2017). Therefore, these substances were targeted in this study. On the contrary, primary aromatic amines were rarely investigated, even though their presence in paper and board FCM can be expected. Indeed, primary aromatic amines can originate from the hydrolysis of aromatic isocyanates in polyurethane adhesives (Pezo et al., 2012). Another source of these substances in paper and board FCM can originate from the degradation of printing azo-dyes or azo-pigments (Yavuz et al., 2016). Consequently, due to their high potential presence in paper and board FCM and their risk to human health (some are carcinogenic), the potential migration of primary aromatic amines was included in the study.

### 3.1 Occurrence of the migratables

First, the potential migration of primary aromatic amines was investigated in accordance with the EURL kitchenware guidelines (Beldi et al., 2023). Out of the 25 targeted primary aromatic amines, only 3,3′-dimethylbenzidine (3,3′-DMB) was found in three takeaway samples (i.e., one pizza box, one noodle box, and one hamburger box) with concentrations ranging from 0.00032 mg kg^-1^ to 0.00052 mg kg^-1^. According to the harmonized classification and labelling (CLP), this substance may cause cancer (Carc.1B) (European chemicals agency, 2023). The presence of this substance can originate from different sources. It can be used as an intermediate for producing azo dyes and insoluble pigments in the paper industry or plastic coatings (Ataman Chemicals, 2023). Noodle and hamburger boxes were coated with polylactic acid, a 100% biodegradable coating, while no information indicated a coating for the pizza box or any recycled fibers. However, the pizza box was coloured due to inscriptions made with inks which could explain the presence of this primary aromatic amine. Due to the absence of harmonized legislation at the European level, the technical guide of the Council of Europe, on paper and board used in food contact materials and articles was applied to evaluate the results (EDQM, 2021). This technical guide lists specific migration limits (SML) for some constituents or contaminants of paper and board FCM, declaring that 1A/1B Classified primary aromatic amines should not be detected in paper and board FCM with a detection limit of 0.002 mg kg^-1^. The concentrations found in this study were lower than this limit.

Although the migration of primary aromatic amines has been well studied in plastic FCM (Brede and Skjevrak, 2004; Trier et al., 2010; Pezo et al., 2012; Merkel et al., 2018), this research is the first to investigate these substances in straws and takeaway articles made from paper and board.

Next, the migration of bisphenols was also observed. Five different substances were targeted in this study (BPA, BPS, BPF, BPZ, and BPB). Only BPA and BPS were detected in 11 takeaway articles. Like primary aromatic amines, bisphenols were not present in straws. An overview of the results in takeaway articles is given in Table 3. An overview of the LOQs is given in Supplementary Table S1.

**TABLE 3:** Concentrations of BPA and BPS in takeaway articles.

	Sample	Concentration in mg kg^−1^	
		BPA	BPS
Boxes	TA-01 Pizza box	0.019	0.015
	TA-02 Pizza box	0.014	<LOQ
	TA-03 Pizza box	0.008	0.017
	TA-48 Pizza box^a^	<LOQ	0.012
	TA-05 Noodle box	0.005	0.016
	TA-25 Hamburger box	0.009	<LOQ
	TA-54 Hamburger box^a^	0.005	0.008
	TA-56 Hamburger box^a^	0.006	0.012
Trays, cones	TA-15 Fries tray	0.026	0.013
	TA-51 Fries tray^a^	<LOQ	0.010
	TA-17 Fries cones	0.009	<LOQ

^a^ Samples were taken in fast food restaurants.

The presence of BPA and BPS in paper and board can originate from different sources like recycling (Almeida et al., 2018), coatings (Lambr et al., 2023), or dyeing of the samples. BPA can also be used as a color developer in thermal paper. After heating, BPA reacts with a leuco dye and changes it to a colored form (Frankowski et al., 2020). None of the samples with BPA or BPS contained a plastic coating, which could explain their presence. For samples taken in fast food restaurants (indicated with * in Table 3), no information on any coating, recycled fiber etc. was available. On the contrary, TA-15 and TA-25 were made of recycled fibers.

According to the last EFSA Scientific opinion (2023), BPA is likely to have immunotoxicity, neurotoxicity, reprotoxic properties as well as developmental toxicity (Lambré et al., 2023). Bisphenol S properties of concern are still under investigation. According to the European chemical agency (ECHA), a majority of data submitters agreed that this substance is toxic to reproduction. The endocrine-disrupting property is still under assessment. Still, according to the latest EFSA opinion on BPA, the TDI has been lowered to 0.2 ng/kg bw/day (Lambr et al., 2023). However, the legislative framework has not yet been updated. Regulation (EU) 2018/213 has set a specific migration limit of 0.05 mg kg^-1^ of food for BPA from varnishes and coatings intended to come into contact with food (COMMISSION REGULATION, 2018). The same SML is mentioned in the technical guide on paper and board of the Council of Europe (EDQM, 2021) and the Regulation (EU) 10/2011 on plastics (Commission Regulation, 2011). For BPS, an SML is only indicated in Regulation (EU) 10/2011 for plastics with the same limit as BPA, i.e. 0.05 mg kg^-1^. All detections of BPA and BPS were well below the SML of 0.05 mg kg^-1^.

Overall, data gaps exist regarding the potential presence of bisphenols in straws and takeaways articles. Suciu et al. investigated the presence of BPA in boxes for frozen pizza and takeaway pizza (Suciu et al., 2013). Although the samples analysed by Suciu et al. are very similar to the samples in the present study, the comparison of the results is hampered by significant differences in the experimental set-up (e.g., extraction with EtOH by Suciu et al. vs. extraction with water (EN 645) in the present study) and the expression of the results (e.g., mg/kg FCM or mg/dm^2^ by Suciu et al. *versus* mg/kg food in the present study).

The presence of photoinitiators could also be expected. Out of the 20 photoinitiators targeted, only benzophenone (BP) and 1-hydroxylcyclohexyl phenylketone (HCPK) were found in 7 takeaway articles, and HCPK was present in one straw. An overview of the results is presented in Table 4. An overview of the LOQs is given in Supplementary Table S1.

**TABLE 4:** Concentrations of BP and HCPK in the straw and takeaway articles.

	Sample	Concentration in mg kg^−1^	
		BP	HCPK
Straws	ST-08 Straw	<LOQ	0.021
Boxes	TA-01 Pizza box	0.006	<LOQ
	TA-02 Pizza box	0.003	<LOQ
	TA-50 Hamburger box *	0.005	0.003
	TA-54 Hamburger box*	0.002	<LOQ
Trays, bags	TA-15 Fries tray	0.005	<LOQ
	TA-51 Fries tray*	0.002	0.007
	TA-57 Fries bag*	<LOQ	0.014

Photoinitiators are used in the UV curing processes of inks and lacquers applied to the packaging surface, mainly cardboard boxes and multilayers. Due to their volatility, these substances can migrate from the packaging and contaminate the food. The majority of samples in which PIs were found were coloured. For the uncoloured samples, the presence could originate from the recycled fibers. However, no information was available.

The technical guide on paper and board of the Council of Europe (EDQM, 2021) referred to the SML set in Regulation (EU) 10/2011 for plastics (Commission Regulation, 2011) and the Swiss Ordinance (SR 817.023.21) (Federal Department of Home Affairs FDHA FFS and VOF, 2020). In both documents, an SML was set at 0.6 mg kg^-1^ for BP, while an SML of 0.01 mg kg^-1^ was set for HCPK in the Swiss Ordinance. All results were well below these limits.

Next, the migration of plasticizers was investigated. Three plasticizers (DBP, DiBP, and DEHP) were present in straws and takeaway articles. Additionally, four more plasticizers (BBP, DIDP, DINP, and DINCH) were detected in the takeaway articles. 60% of the straws contained at least one phthalate compared to 56% for takeaway articles. However, more plasticizers were found in takeaway articles. As plasticizers were found in many samples, Table 5 gives an overview of the results (e.g., minimum, maximum, and median concentrations). A detailed overview of the results is available in Supplementary Table S8. An overview of the LOQs is given in Supplementary Table S1.

**TABLE 5:** Concentration of plasticizers in takeaway articles and straws expressed in mg kg^−1^, minimum, maximum concentrations and median, count of samples with detection above the LOQ (n) and percentage of sample in which the compound was quantified.

Substances	n	% of samples containing the phthalate	Minimum (mg/kg)	Maximum (mg/kg)	Median (mg/kg)	Sample type with highest concentration
Takeaway articles						
DiBP	28	36	0.006	0.46	0.017	Takeaway Box
DBP	19	24	0.005	0.07	0.025	Noodle box
DEHP	21	27	0.005	0.15	0.039	Pizza box
DiDP	1	1	0.01	Coffee cup		
DiNP	19	24	0.012	0.12	0.035	Noodle box
DINCH	14	18	0.006	0.011	0.023	Hamburger box
BBP	10	13	0.005	0.013	0.006	Fries cone
Straws						
DiBP	10	13	0.008	0.029	0.013	White and red straw
DBP	5	7	0.007	0.032	0.015	White and red straw
DEHP	9	12	0.011	0.049	0.041	Bicolor straw

In the FCM industry, plasticizers can increase the flexibility of plastic materials and can also be part of printing inks and lacquers. In addition to printing inks or lacquers used in paper and board FCM, the recycling process associated with/or the environmental contamination could explain the presence of these substances. Notwithstanding, although plasticizers have exceptional properties, these substances can pose a risk to human health. Indeed, according to the CLP (Classification, Labelling and Packaging) classification, DBP, DiBP, BBP, and DEHP are toxic to reproduction (Repro 1B) and endocrine disruptors. The SMLs in the technical guide on paper and board of the Council of Europe (EDQM, 2021) refer to Regulation (EU) 10/2011 (Commission Regulation, 2011) stipulating an SML of 1.5 mg kg^-1^ for DEHP. Additionally, the sum of DBP and DiBP should not exceed 0.3 mg kg^-1^. Based on these SMLs, no straw exceeded the limits. On the contrary, the sum of DiBP and DBP exceeded this limit in one takeaway box with 0.46 mg kg^-1^. Regarding BBP, DINP, DIDP, and DINCH, Regulation (EU) 10/2011 and the Swiss Ordinance set a specific migration limit of 30 mg kg^-1^ for BBP, 9 mg kg^-1^ for the sum of DIDP and DINP and 60 mg kg^-1^ for DINCH. None of these SMLs were exceeded. Overall, the comparison to SML should be qualified as there are usage restrictions for phthalates in the EU Regulation 10/2011 (e.g., the substance can only be used in repeated use articles containing non-fatty foods), which are not included in the EDQM document. Based on these restrictions, the interpretation could be different. It should be noted that Regulation (EU) 10/2011 has recently been amended (COMMISSION REGULATION, 2023), thereby changing the restrictions for phthalates. However, the sampling was conducted before the updated regulation entered into force. The results were thus evaluated according to the applicable legislation at the time of the sampling.

When comparing the results to other studies, it was observed that plasticizers are frequently found in paper and board FCM. A study conducted in 2007 already showed the presence of DiBP in 16 takeaway pizza boxes (Bononi and Tateo, 2009). The same year, another study showed the presence of DBP and DEHP in takeaway items (pizza, fries bags etc.) (Lopez-Espinosa et al., 2007). However, the methodologies applied in these studies were not comparable to the current study, and, therefore, the observed concentrations cannot be compared. More recently, in 2013, Suciu et al. found DEHP in 50% of the pizza boxes analyzed in their study (Suciu et al., 2013).

Finally, mineral oil saturated hydrocarbons (MOSH) and mineral oil aromatic hydrocarbons (MOAH) fractions from C10 to C50 were investigated in 2 straws and 53 takeaway articles. Not all the samples were analyzed as it was not expected to find mineral oil hydrocarbons in straws. However, MOSH and MOAH were found in the two straws with an average concentration of MOSH of 26.3 mg kg^-1^ and 0.32 mg kg^-1^ of MOAH.

All the analyzed takeaway articles contained MOSH, while MOAH was found in 88.7% of takeaway articles. The results per sample are available in Supplementary Table S9. Table 6 gives an overview of the results (e.g., minimum, maximum, and median concentrations).

**TABLE 6:** Concentration of mineral oil in takeaway articles and straws expressed in mg kg^-1^, minimum, maximum concentrations and median, count of samples with detection above the LOQ (n) and percentage of sample in which the compound was quantified.

Substances	n	% of samples containing the MOH	Minimum (mg/kg)	Maximum (mg/kg)	Median (mg/kg)	Sample type with highest concentration
Takeaway articles						
MOSH	53	100	0.01	35.9	0.83	Coffee cup
MOAH	47	88.7	0.01	1.76	0.16	Noodle box
Straws						
MOSH	2	100	1.5	51.0	26.2	Black Paper straw for cold beverages
MOAH	2	100	0.05	0.59	0.3	

Mineral oil hydrocarbons can potentially impact human health depending on their structure. Aromatic mineral oils can act as genotoxic carcinogens, while saturated mineral oils can accumulate in human tissues (EFSA Panel on Contaminants in the Food Chain (CONTAM), 2012). Consequently, in April 2022, the European Commission proposed action limits for MOAHs in all foodstuffs as sold, regardless of the contamination source (packaging materials or other sources) (European commission, 2022). These limits were set at 0.5 mg/kg for dry foods with a low fat/oil content (≤4% fat/oil), 1 mg/kg for foods with a higher fat/oil content (>4% fat/oil) and 2 mg/kg for fats/oils.

Assuming 100% of migration from the food contact material into food, one straw (ST-04), one pizza box (TA-01), one noodle box (TA-05), and three cups (TA-34, TA-38 and TA-40) exceeded their associated limits of 0.5 mg kg^-1^ for the straw and cups and 1 mg kg^-1^ for pizza and noodle boxes. Regarding MOSH, the Scientific Committee of the Belgian Federal Agency for the Safety of the Food Chain proposed action limits in different food categories based on FoodEx level 1 consumption data for kids (Scicom, 2017). Still assuming 100% of migration, the limit of 10 mg kg^-1^ of MOSH was exceeded for one straw (ST-04) and two coffee cups (TA-34, TA-40).

The content of MOSH and MOAH in paper and board has already been well investigated for dry food sold on the market (Vollmer et al., 2011; Grob, 2018). However, limited data exist on takeaway articles or straws. Fengler et al. (2015) studied the content of MOSH and MOAH in fast food packaging (Fengler et al., 2015) and Conchione et al. (2020) in pizza boxes. To allow comparison with the current study, the results of the present study were converted in mg kg^-1^ of paper and board (Table 7).

**TABLE 7:** Minimum and maximum concentrations of MOSH and MOAH in takeaway articles expressed in mg kg^−1^ of paper and board.

Sample type		Concentration in mg kg^-1^ of paper and board			
		MOSH		MOAH	
	n	Min	Max	Min	Max
Pizza boxes	4	136.6	409.2	28.0	128.8
Hamburger boxes	8	26.0	331.2	3.8	87.5
Fries trays/bags	7	6.3	324.4	1.4	61.1
Wraps	6	17.4	32.45	0.83	2.8

In the study of Fengler et al. (2015), the content in pizza boxes ranged from 19 to 682 mg kg^-1^ of paper and board for MOSH, while the MOAH content ranged from 9 to around 90 mg kg^-1^ paper and board. Only one pizza box had a low level of MOSH and MOAH in the study of Fengler et al. (2015), while the three others were comparable between both studies. In hamburger boxes, fries trays, and a wrap, Fengler et al. (2015) found MOAH concentrations below 40 mg kg^-1^ of paper and board and around 120 mg kg^-1^ of paper and board for MOSH except for one fries tray with concentrations of MOSH of 511 mg kg^-1^ and 92 mg kg^-1^ of MOAH. The difference in concentration in the other categories could be explained by a higher variability of samples found on the market. As only a limited number of samples have been analyzed in the study of Fengler et al. (2015), a comparison of results would be biased.

In the study of Conchione et al. (2020), samples were divided in two categories. Category I groups pizza boxes potentially made of virgin paper board while group II groups samples suspected to contain recycled fibers. The analyses of group I showed the lowest concentrations of MOSH and MOAH compared to group II with MOSH concentration from fractions C10−35 ranging from 3.2 to 68.8 mg kg^-1^ paper and board with an average amount of 8.5 mg kg^-1^ paper and board. MOAH (C10−35) levels were most of the time below 5 mg kg^-1^ paper and board. On the contrary, samples belonging to group II showed higher level of MOSH and MOAH with concentration of MOSH between 123.7 up to 650.3 mg kg^-1^ paper and board with an average amount of 332.8 mg kg^-1^ paper and board. Regarding MOAH, concentrations ranged from 9.8 up to 70.3 mg kg^-1^ paper and board with an average of 33.3 mg kg^-1^ paper and board. In the present study, the results obtained for pizza boxes are more similar to the results of group II, potentially indicating that recycled fibers were present in the samples.

### 3.2 Risk assessment of migratables

Although new FCMs can be innovative and environmentally friendly, they should also be safe for consumers. However, contaminants of concern can still be present, potentially migrating into food. Since no specific EU legislation exists for paper and board, a risk assessment was performed on all migrants. Based on the migration results and considering the exposure scenarios, the potential risks were assessed for children, teenagers and adults. Table 8 presents the categories of samples and substances of potential concern for consumers.

**TABLE 8:** Overview of the results of the risk assessment.

	BPA			3,3-DMB			MOSH		
Samples	Children	Teenagers	Adults	Children	Teenagers	Adults	Children	Teenagers	Adults
Pizza box	Potential risk			Potential risk		Low probability of adverse health effect	Low concern for public health		
Noodle box	Potential risk			Low probability of adverse health effect			Low concern for public health		
Fries tray, bag	Potential risk			Low probability of adverse health effect			Low concern for public health		
Hamburger box	Potential risk			Low probability of adverse health effect			Low concern for public health		
Takeaway Box	No risk			Low probability of adverse health effect			Low concern for public health		
Straw	No risk			Low probability of adverse health effect			Potential risk	Low concern for public health	
Paper spoon	No risk			Low probability of adverse health effect			Low concern for public health		
Cup	No risk			Low probability of adverse health effect			Low concern for public health		
Snack tray	No risk			Low probability of adverse health effect			Low concern for public health		
Sandwich bag, wrap	No risk			Low probability of adverse health effect			Low concern for public health		
Ice cream bowl	No risk			Low probability of adverse health effect			Low concern for public health		
Bowl	No risk			Low probability of adverse health effect			Low concern for public health		

Samples	MOAH (10%)					MOAH (1% scenario)			
	Children	Teenagers			Adults	Children	Teenagers	Adults	
Pizza box	Potential risk					Low concern for public health			
Noodle box	Potential risk					Low concern for public health			
Fries tray, bag	Potential risk					Low concern for public health			
Hamburger box	Potential risk					Low concern for public health			
Takeaway Box	Low concern for public health	Potential risk	Low concern for public health			Low concern for public health			
Straw	Potential risk		Low concern for public health			Potential risk	Low concern for public health		
Paper spoon	Potential risk					Low concern for public health			
Cup	Potential risk					Low concern for public health	Potential risk		Low concern for public health
Snack tray	Low concern for public health	Potential risk				Low concern for public health			
Sandwich bag, wrap	Low concern for public health					Low concern for public health			
Ice cream bowl	Low concern for public health	Potential risk		Low concern for public health		Low concern for public health			
Bowl	Low concern for public health					Low concern for public health			

Overall, the results brought to light potential concerns for consumers for bisphenol A, 3.3′DMB, and mineral oil hydrocarbons and this for several sample categories. According to the latest opinion of the EFSA panel on MOH, higher exposure was highlighted for children (Schrenk et al., 2023). The same trend is only highlighted for one sample (a straw) regarding MOSH. The Panel concluded that the present dietary exposure to MOSH does not raise concern for human health for all population categories considering a margin of exposure approach (Schrenk et al., 2023). Except for one straw, this statement is in accordance with the conclusion of the EFSA Panel.

Regarding MOAH, two scenarios were considered in accordance with the latest scientific opinion (Schrenk et al., 2023). These two scenarios consider the BMDL10 of 0.49 mg kg^-1^ bw per day, with average contents of 10% or 1% of 3- or more ring MOAH within the MOAH fraction, since this sub-fraction is considered the most toxic (Schrenk et al., 2023). When it is considered that the result obtained contains 10% MOAH with 3 rings or more, 10 categories of samples out of 12 are a potential risk for consumers. On the other hand, when only 1% of the MOAH fraction would consist of 3 or more rings, only 2 categories of samples remain at potential risk for consumers. Nonetheless, it should be noted that the obtained results can be considered a worst-case since they were based on extraction rather than migration, potentially overestimating the actual migration into food. Therefore, it would be relevant to analyze the food itself (e.g., a pizza instead of a pizza box) or using a food simulant, if possible, to conduct a more realistic risk assessment.

## 4 Conclusion

Seventy-eight food contact materials made of paper and board were analyzed to identify potential migrations of harmful substances to human health, such as plasticizers, bisphenols, photoinitiators, mineral oil, and primary aromatic amines. The extraction experiments highlighted the presence of 14 substances out of the 66 targeted in the samples. Photoinitators were detected in 9% of the samples, bisphenols in 13%, primary aromatic amines in 5%, plasticizers in 56%, and mineral oil in 100%. Overall, few results exceeded their associated limits. One takeaway box exceeded the limit for DiBP, while six samples (straw, cups, noodle and pizza boxes) exceeded their associated limit for MOAH and three samples (straw and cups) for MOSH. A risk assessment was carried out for each migrant, highlighting a potential concern for the consumer for four substances in several FCM categories. Although the methods are already well-established, the simultaneous application of these methods allows to obtain an overview of the mixtures to which the consumer is exposed. The potential risks related to these mixtures could be assessed in the future, which is more realistic than the risk assessment related to exposure to individual substances. This study also demonstrates the importance of further and more realistic evaluation of these materials, in particular by migration tests or analysis of the food itself. Finally, the results of the study can also be used to guide future monitoring programs to ensure the safety of the consumer.

## Data Availability

The original contributions presented in the study are included in the article/Supplementary Material, further inquiries can be directed to the corresponding author.
