# Supplementary material for: Investigation of potential migratables from paper and board food contact materials
Source: Front Chem. 2023 Nov 30;11:1322811. doi: 10.3389/fchem.2023.1322811 (PMC10720245; doi:10.3389/fchem.2023.1322811)
Supplement: Supplementary file 1 [file Table1.docx]

**SUPPLEMENTARY DATA**

**Table S1: Limit of quantification per sample and category of substances**

| **Sample ID** | **Bisphenols** | **Primary aromatic amines** | **Photoinitiators** | **Plasticizers** |
| --- | --- | --- | --- | --- |
| **ST-01** | 0.0094 | 0.0005 | 0.0047 | 0.009 |
| **ST-02** | 0.0091 | 0.0005 | 0.0045 | 0.009 |
| **ST-03** | 0.0068 | 0.0003 | 0.0034 | 0.007 |
| **ST-04** | 0.0150 | 0.0008 | 0.0075 | 0.015 |
| **ST-05** | 0.0095 | 0.0005 | 0.0047 | 0.009 |
| **ST-06** | 0.0097 | 0.0005 | 0.0048 | 0.010 |
| **ST-07** | 0.0097 | 0.0005 | 0.0049 | 0.010 |
| **ST-08** | 0.0096 | 0.0005 | 0.0048 | 0.010 |
| **ST-09** | 0.0097 | 0.0005 | 0.0049 | 0.010 |
| **ST-10** | 0.0058 | 0.0003 | 0.0029 | 0.006 |
| **ST-11** | 0.0076 | 0.0004 | 0.0038 | 0.008 |
| **ST-12** | 0.0081 | 0.0004 | 0.0040 | 0.008 |
| **ST-13** | 0.0088 | 0.0004 | 0.0044 | 0.009 |
| **ST-14** | 0.0099 | 0.0005 | 0.0049 | 0.010 |
| **ST-15** | 0.0169 | 0.0009 | 0.0085 | 0.017 |
| **ST-16** | 0.0086 | 0.0004 | 0.0043 | 0.009 |
| **ST-17** | 0.0080 | 0.0004 | 0.0040 | 0.008 |
| **ST-18** | 0.0080 | 0.0004 | 0.0040 | 0.008 |
| **ST-19** | 0.0080 | 0.0004 | 0.0040 | 0.008 |
| **ST-20** | 0.0114 | 0.0006 | 0.0057 | 0.011 |
| **TA-01** | 0.0045 | 0.0002 | 0.0023 | 0.005 |
| **TA-02** | 0.0045 | 0,0002 | 0.0023 | 0.005 |
| **TA-03** | 0.0045 | 0.0002 | 0.0023 | 0.005 |
| **TA-04** | 0.0045 | 0.0002 | 0.0023 | 0.005 |
| **TA-05** | 0.0045 | 0.0002 | 0.0023 | 0.005 |
| **TA-06** | 0.0045 | 0.0002 | 0.0023 | 0.005 |
| **TA-07** | 0.0045 | 0.0002 | 0.0023 | 0.005 |
| **TA-08** | 0.0045 | 0.0002 | 0.0023 | 0.005 |
| **TA-09** | 0.0045 | 0.0002 | 0.0023 | 0.005 |
| **TA-10** | 0.0090 | 0.0005 | 0.0045 | 0.009 |
| **TA-11** | 0.0090 | 0.0005 | 0.0045 | 0.009 |
| **TA-12** | 0.0090 | 0.0005 | 0.0045 | 0.009 |
| **TA-13** | 0.0090 | 0.0005 | 0.0045 | 0.009 |
| **TA-14** | 0.0045 | 0.0002 | 0.0023 | 0,005 |
| **TA-15** | 0.0045 | 0.0002 | 0.0023 | 0.005 |
| **TA-16** | 0.0045 | 0.0002 | 0.0023 | 0.005 |
| **TA-17** | 0.0045 | 0.0002 | 0.0023 | 0.005 |
| **TA-18** | 0.0127 | 0.0006 | 0.0063 | 0.013 |
| **TA-19** | 0.0130 | 0.0007 | 0.0065 | 0.013 |
| **TA-20** | 0.0045 | 0.0002 | 0.0023 | 0.005 |
| **TA-21** | 0.0045 | 0.0002 | 0.0023 | 0.005 |
| **TA-22** | 0.0045 | 0.0002 | 0.0023 | 0.005 |
| **TA-23** | 0.0045 | 0.0002 | 0.0023 | 0.005 |
| **TA-24** | 0.0045 | 0.0002 | 0.0023 | 0.005 |
| **Sample ID** | **Bisphenols** | **Primary aromatic amines** | **Photoinitiators** | **Plasticizers** |
| **TA-25** | 0.0045 | 0.0002 | 0.0023 | 0.005 |
| **TA-26** | 0.0045 | 0.0002 | 0.0023 | 0.005 |
| **TA-27** | 0.0080 | 0.0004 | 0.0040 | 0.008 |
| **TA-28** | 0.0111 | 0.0006 | 0.0056 | 0.011 |
| **TA-29** | 0.0119 | 0.0006 | 0.0059 | 0.012 |
| **TA-30** | 0.0096 | 0.0005 | 0.0048 | 0.010 |
| **TA-31** | 0.0080 | 0.0004 | 0.0040 | 0.008 |
| **TA-32** | 0.0091 | 0.0005 | 0.0046 | 0.009 |
| **TA-33** | 0.0090 | 0.0005 | 0.0045 | 0.011 |
| **TA-34** | 0.0067 | 0.0003 | 0.0033 | 0.003 |
| **TA-35** | 0.0081 | 0.0004 | 0.0041 | 0.008 |
| **TA-36** | 0.0083 | 0.0004 | 0.0041 | 0.008 |
| **TA-37** | 0.0089 | 0.0005 | 0.0045 | 0.009 |
| **TA-38** | 0.0094 | 0.0005 | 0.0047 | 0.005 |
| **TA-39** | 0.0079 | 0.0004 | 0.0039 | 0.005 |
| **TA-40** | 0.0066 | 0.0003 | 0.0033 | 0.005 |
| **TA-41** | 0.0080 | 0.0004 | 0.0040 | 0.005 |
| **TA-42** | 0.0094 | 0.0005 | 0.0047 | 0.005 |
| **TA-43** | 0.0045 | 0.0002 | 0.0023 | 0.005 |
| **TA-44** | 0.0086 | 0.0004 | 0.0043 | 0.005 |
| **TA-45** | 0.0086 | 0.0004 | 0.0043 | 0.009 |
| **TA-46** | 0.0045 | 0.0002 | 0.0023 | 0.005 |
| **TA-47** | 0.0045 | 0.0002 | 0.0023 | 0.005 |
| **TA-48** | 0.0045 | 0.0002 | 0.0023 | 0.005 |
| **TA-49** | 0.0045 | 0.0002 | 0.0023 | 0.005 |
| **TA-50** | 0.0045 | 0.0002 | 0.0023 | 0.005 |
| **TA-51** | 0.0045 | 0.0002 | 0.0023 | 0.005 |
| **TA-52** | 0.0045 | 0.0002 | 0.0023 | 0.005 |
| **TA-53** | 0.0045 | 0.0002 | 0.0023 | 0.005 |
| **TA-54** | 0.0045 | 0.0002 | 0.0023 | 0.005 |
| **TA-55** | 0.0045 | 0.0002 | 0.0023 | 0.005 |
| **TA-56** | 0.0045 | 0.0002 | 0.0023 | 0.005 |
| **TA-57** | 0.0045 | 0.0002 | 0.0023 | 0.005 |
| **TA-58** | 0.0096 | 0.0005 | 0.0048 | 0.010 |
