# Supplementary material for: Investigation of potential migratables from paper and board food contact materials
Source: Front Chem. 2023 Nov 30;11:1322811. doi: 10.3389/fchem.2023.1322811 (PMC10720245; doi:10.3389/fchem.2023.1322811)
Supplement: Supplementary file 2 [file Table8.docx]

**SUPPLEMENTARY DATA**

***Table S8: Occurrences of plasticizers in straws and takeaway articles in mg kg^-1^***

| **Sample ID** | | **Concentrations expressed in mg kg-1** | | | | | | | |  |  |  |  |  |  |
| --- | --- | --- | --- | --- | --- | --- | --- | --- | --- | --- | --- | --- | --- | --- | --- |
|  |  | **DBP** | **DiBP** | **BBP** | **DEHP** | **DIDP** | **DINP** | **DINCH** |  |  |  |  |  |  |  |
| **ST - 01** | - | | - | - | - | - | - | - |  |  |  |  |  |  |  |
| **ST - 02** | - | | - | - | - | - | - | - |  |  |  |  |  |  |  |
| **ST - 03** | - | | 0.008 | - | - | - | - | - |  |  |  |  |  |  |  |
| **ST - 04** | - | | - | - | 0.041 | - | - | - |  |  |  |  |  |  |  |
| **ST - 05** | - | | 0.012 | - | 0.043 | - | - | - |  |  |  |  |  |  |  |
| **ST - 06** | 0.032 | | 0.029 | - | 0.042 | - | - | - |  |  |  |  |  |  |  |
| **ST - 07** | 0.023 | | 0.016 | - | 0.035 | - | - | - |  |  |  |  |  |  |  |
| **ST - 08** | 0.014 | | 0.017 | - | 0.030 | - | - | - |  |  |  |  |  |  |  |
| **ST - 09** | 0.015 | | 0.024 | - | 0.049 | - | - | - |  |  |  |  |  |  |  |
| **ST - 10** | 0.007 | | 0.011 | - | 0.011 | - | - | - |  |  |  |  |  |  |  |
| **ST - 11** | - | | 0.008 | - | - | - | - | - |  |  |  |  |  |  |  |
| **ST - 12** | - | | 0.013 | - | - | - | - | - |  |  |  |  |  |  |  |
| **ST - 13** | - | | 0.010 | - | 0.011 | - | - | - |  |  |  |  |  |  |  |
| **ST - 14** | - | | - | - | - | - | - | - |  |  |  |  |  |  |  |
| **ST - 15** | - | | - | - | - | - | - | - |  |  |  |  |  |  |  |
| **ST - 16** | - | | - | - | - | - | - | - |  |  |  |  |  |  |  |
| **ST - 17** | - | | - | - | - | - | - | - |  |  |  |  |  |  |  |
| **ST - 18** | - | | - | - | - | - | - | - |  |  |  |  |  |  |  |
| **ST - 19** | - | | - | - | - | - | - | - |  |  |  |  |  |  |  |
| **ST - 20** | - | | - | - | 0.040 | - | - | - |  |  |  |  |  |  |  |
| **TA - 01** | 0.032 | | 0.024 | 0.005 | 0.152 | - | 0.088 | 0.012 |  |  |  |  |  |  |  |
| **TA - 02** | 0.036 | | 0.042 | 0.012 | 0.121 | - | 0.085 | 0.020 |  |  |  |  |  |  |  |
| **TA - 03** | 0.028 | | 0.027 | 0.006 | 0.073 | - | 0.059 | 0.059 |  |  |  |  |  |  |  |
| **TA - 04** | - | | - | - | - | - | - | - |  |  |  |  |  |  |  |
| **TA - 05** | 0.073 | | 0.063 | - | 0.138 | - | 0.119 | 0.037 |  |  |  |  |  |  |  |
| **TA - 06** | - | | - | - | - | - | - | - |  |  |  |  |  |  |  |
| **TA - 07** | - | | - | - | - | - | - | - |  |  |  |  |  |  |  |
| **TA - 08** | - | | - | - | - | - | - | - |  |  |  |  |  |  |  |
| **TA - 09** | - | | - | - | - | - | - | - |  |  |  |  |  |  |  |
| **TA - 10** | - | | - | - | - | - | - | - |  |  |  |  |  |  |  |
| **TA - 11** | - | | - | - | - | - | - | - |  |  |  |  |  |  |  |
| **TA - 12** | - | | - | - | - | - | - | - |  |  |  |  |  |  |  |
| **TA - 13** | - | | - | - | - | - | - | 0.035 |  |  |  |  |  |  |  |
| **TA - 14** | 0.005 | | 0.008 | - | - | - | - | - |  |  |  |  |  |  |  |
| **TA - 15** | 0.043 | | 0.044 | 0.009 | 0.146 | - | 0.066 | 0.022 |  |  |  |  |  |  |  |
| **TA - 16** | 0.009 | | 0.012 | - | 0.008 | - | 0.014 | - |  |  |  |  |  |  |  |
| **TA - 17** | 0.034 | | 0.030 | 0.013 | 0.124 | - | 0.116 | 0.025 |  |  |  |  |  |  |  |
| **TA - 18** | - | | 0.017 | - | - | - | - | - |  |  |  |  |  |  |  |
| **TA - 19** | - | | 0.015 | - | - | - | - | - |  |  |  |  |  |  |  |
| **TA - 20** | 0.007 | | 0.007 | - | - | - | - | - |  |  |  |  |  |  |  |
| **TA - 21** | - | | 0.457 | - | - | - | - | - |  |  |  |  |  |  |  |
| **TA - 22** | - | | - | - | - | - | - | - |  |  |  |  |  |  |  |
| **TA - 23** | - | | 0.009 | - | 0.011 | - | 0.012 | - |  |  |  |  |  |  |  |
| **TA - 24** | - | | 0.006 | - | 0.005 | - | - | - |  |  |  |  |  |  |  |
| **TA - 25** | 0.012 | | 0.020 | 0.010 | 0.042 | - | 0.023 | 0.112 |  |  |  |  |  |  |  |
| **TA - 26** | 0.009 | | 0.016 | - | 0.009 | - | 0.028 | - |  |  |  |  |  |  |  |
| **TA - 27** | **-** | | | | | | | | - | | - | - | - | - | - |
| **TA - 28** | **-** | | **-** | **-** | **-** | **-** | **-** | **-** |  |  |  |  |  |  |  |
| **TA - 29** | - | | - | - | - | - | - | - |  |  |  |  |  |  |  |
| **TA - 30** | - | | - | - | - | - | - | - |  |  |  |  |  |  |  |
| **TA - 31** | - | | - | - | - | - | - | - |  |  |  |  |  |  |  |
| **TA - 32** | - | | - | - | - | - | - | - |  |  |  |  |  |  |  |
| **TA - 33** | 0.033 | | 0.037 | - | 0.021 | - | 0.024 | - |  |  |  |  |  |  |  |
| **TA - 34** | - | | - | - | 0.005 | - | - | - |  |  |  |  |  |  |  |
| **TA - 35** | - | | - | - | - | - | - | - |  |  |  |  |  |  |  |
| **TA - 36** | - | | - | - | - | - | - | - |  |  |  |  |  |  |  |
| **TA - 37** | - | | - | - | - | - | - | - |  |  |  |  |  |  |  |
| **TA - 38** | 0.026 | | 0.020 | - | 0.056 | - | 0.020 | - |  |  |  |  |  |  |  |
| **TA - 39** | - | | - | - | - | - | - | - |  |  |  |  |  |  |  |
| **TA - 40** | - | | 0.011 | - | - | - | - | - |  |  |  |  |  |  |  |
| **TA - 41** | - | | - | - | - | - | - | - |  |  |  |  |  |  |  |
| **TA - 42** | - | | - | - | 0.006 | 0.010 | 0.024 | - |  |  |  |  |  |  |  |
| **TA - 43** | - | | - | - | - | - | - | - |  |  |  |  |  |  |  |
| **TA - 44** | - | | - | - | - | - | - | - |  |  |  |  |  |  |  |
| **TA - 45** | - | | - | - | - | - | - | - |  |  |  |  |  |  |  |
| **TA - 46** | - | | - | - | - | - | - | - |  |  |  |  |  |  |  |
| **TA - 47** | - | | - | - | - | - | - | - |  |  |  |  |  |  |  |
| **TA - 48** | 0.029 | | 0.022 | 0.006 | 0.084 | - | 0.056 | 0.012 |  |  |  |  |  |  |  |
| **TA - 49** | - | | 0.006 | - | - | - | - | - |  |  |  |  |  |  |  |
| **TA - 50** | 0.016 | | 0.017 | 0.005 | 0.032 | - | 0.035 | 0.021 |  |  |  |  |  |  |  |
| **TA - 51** | 0.015 | | 0.016 | 0.006 | 0.035 | - | 0.044 | 0.023 |  |  |  |  |  |  |  |
| **TA - 52** | - | | 0.012 | - | - | - | - | - |  |  |  |  |  |  |  |
| **TA - 53** | 0.011 | | 0.013 | - | - | - | - | - |  |  |  |  |  |  |  |
| **TA - 54** | 0.025 | | 0.047 | - | 0.091 | - | 0.034 | 0.006 |  |  |  |  |  |  |  |
| **TA - 55** | - | | - | - | - | - | - | - |  |  |  |  |  |  |  |
| **TA - 56** | 0.025 | | 0.025 | 0.006 | 0.039 | - | 0.050 | 0.010 |  |  |  |  |  |  |  |
| **TA - 57** | 0.006 | | 0.007 | - | 0.023 | - | 0.018 | 0.028 |  |  |  |  |  |  |  |
| **TA - 58** | - | | - | - | - | - | - | - |  |  |  |  |  |  |  |
