# Supplementary material for: Investigation of potential migratables from paper and board food contact materials
Source: Front Chem. 2023 Nov 30;11:1322811. doi: 10.3389/fchem.2023.1322811 (PMC10720245; doi:10.3389/fchem.2023.1322811)
Supplement: Supplementary file 3 [file Table7.docx]

**SUPPLEMENTARY DATA**

***Table S7: Description of the samples based on the labelling.***

| **SAMPLE ID** | **DESCRIPTION** | **MATERIAL** |
| --- | --- | --- |
| **ST - 01** | Straight Paper straw (BPA, phthalates free) | Paper, no treatment |
| **ST - 02** | Flexible Black paper straw | Paper |
| **ST - 03** | Straight Kraft paper straw | Kraft paper |
| **ST - 04** | Straight Black Paper straw (cold brevage only, compostable) | Paper |
| **ST - 05** | Flexible paper straw paper (resistance up to 70°C) | Paper |
| **ST - 06** | White and red straight paper straw | Paper, edible inks |
| **ST – 07** | Bamboo look straight paper straw (resistance up to 70°C) | Paper |
| **ST – 08** | Blue and white flexible paper straw (cold beverage only, compostable) | Made from plants |
| **ST – 09** | White and yellow straight paper straw | Paper |
| **ST – 10** | Straight black paper straw | Kraft paper |
| **ST – 11** | Flexible color paper straw (resistance up to 70°C) | Paper |
| **ST – 12** | Mojito black straight paper straw (cold brevage only, compostable) | Paper |
| **ST – 13** | Flexible white paper straw (apple juice packaging) | Paper |
| **ST – 14** | Flexible white paper straw (Cécémel packaging) | Paper |
| **ST – 15** | Straight white paper straw (Capri-sun packaging) | Paper |
| **ST – 16** | Flexible white paper straw (multivatamins juice packaging) | Paper |
| **ST – 17** | Flexible white paper straw (Milk packaging) | Paper |
| **ST – 18** | White straw (fast food) | Paper |
| **ST – 19** | Straw | Paper |
| **ST – 20** | Straw | Paper |
| **TA – 01** | Pizza box | Cardboard |
| **TA – 02** | Pizza box | Cardboard |
| **TA – 03** | Pizza box for 1 slice | Cardboard,  no recycled fibers |
| **TA – 04** | Noodle box | Paper with PLA coating |
| **TA – 05** | Noodle box | Plastic-free greasefree cardboard |
| **TA – 06** | Noodle box | Kraft paper with corn starch coating (PLA) |
| **TA – 07** | Noodle box | White Cardboard with polyethylene |
| **TA – 08** | Fries paper cone | Greaseproof paper |
| **TA – 09** | Fries bag | Greaseproof Kraft paper |
| **TA – 10** | Hamburger wrap | Greaseproof paper |
| **TA – 11** | Hamburger wrap | Greaseproof paper |
| **TA – 12** | Hamburger wrap | Sulfurized greaseproof paper |
| **TA – 13** | Paper snack and sandwich bag | Greaseproof paper without waxes or chlorine |
| **TA – 14** | Fries tray | Cardboard |
| **TA – 15** | Fries tray | Kraft Cardboard, contain recycled fibers |
| **TA – 16** | Snack tray | Kraft Cardboard |
| **TA – 17** | Fries cones | Nano micro Kraft paper ; contain recycled fibers |
| **TA – 18** | Paper spoon | Paper |
| **TA – 19** | Paper spoon | Paper |
| **TA – 20** | Takeaway Cardboard Box | Cardboard, water based coating |
| **TA – 21** | Takeaway Box | Cardboard with polyethylene coating |
| **TA – 22** | Hamburger box | Kraft cardboard + PLA coating |
| **TA – 23** | Hamburger box | Non biodegradable cardboard |
| **TA – 24** | Hamburger box | Cardboard, nanotechnology ; greasefree |
| **TA – 25** | Hamburger box | Recycled Cardboard |
| **TA – 26** | Hamburger box | Greaseproof kraft cardboard, unspecified white varnishe |
| **TA – 27** | Soup bowl | Cardboard + PLA coating |
| **TA – 28** | Ice cream bowl | Cardboard |
| **TA – 29** | Bowl | Cardboard |
| **TA – 30** | Soup bowl | Cardboard + PLA coating |
| **TA – 31** | Ice cream bowl | Cardboard + PLA coating |
| **TA – 32** | Bowl | Cardboard |
| **TA – 33** | Coffee cup | Kraft paper + PLA coating |
| **TA – 34** | Coffee cup | Paper + PLA coating |
| **TA – 35** | Cup | Kraft paper + PLA coating |
| **TA – 36** | Coffee cup | Paper + PLA coating |
| **TA – 37** | Soup bowl | Cardboard + unspecified coating |
| **TA – 38** | Cup | Cardboard + PLA coating |
| **TA – 39** | Cup | Paper |
| **TA – 40** | Coffee cup | Cardboard + unspecified plastic coating |
| **TA – 41** | Cup | Cardboard + polyethylene coating |
| **TA – 42** | Coffee cup | Cardboard + unspecified plastic coating |
| **TA – 43** | Cup | Cardboard |
| **TA – 44** | Coffee cup | Cardboard + PLA coating |
| **TA – 45** | Coffee cup | Cardboard + unspecified coating |
| **TA – 46** | Tacos wrap (Fast food) | Paper ; no info on possible coating |
| **TA – 47** | Sushi tray (Fast food) | Cardboard ; no info on possible coating |
| **TA – 48** | Pizza Box (Fast food) | Cardboard |
| **TA – 49** | Hamburger wrap (Fast food) | Paper ; no info on possible coating |
| **TA – 50** | Hamburger box (Fast food) | Cardboard ; no info on possible coating |
| **TA – 51** | Fries tray (Fast food) | Cardboard ; no info on possible coating |
| **TA – 52** | Fries bag (Fast food) | Paper ; no info on possible coating |
| **TA – 53** | Chicken box (Fast food) | Cardboard ; no info on possible coating |
| **TA – 54** | Burger box (Fast food) | Cardboard ; no info on possible coating |
| **TA – 55** | Fries bag (Fast food) | Paper ; no info on possible coating |
| **TA – 56** | Hamburger box (Fast food) | Cardboard ; no info on possible coating |
| **TA – 57** | Fries bag (Fast food) | Paper ; no info on possible coating |
| **TA – 58** | Soda cup (Fast food) | Paper + unspecified plastic coating |
