# Supplementary material for: Investigation of potential migratables from paper and board food contact materials
Source: Front Chem. 2023 Nov 30;11:1322811. doi: 10.3389/fchem.2023.1322811 (PMC10720245; doi:10.3389/fchem.2023.1322811)
Supplement: Supplementary file 4 [file Table9.docx]

**SUPPLEMENTARY DATA**

***Table S9: Occurrences of MOSH and MOAH in straws and takeaway articles in mg kg^-1^***

| **Sample ID** | **Concentrations expressed in mg kg -1** | |
| --- | --- | --- |
|  | **MOSH** | **MOAH** |
| **ST-04** | 51.0 | 0.59 |
| **ST-11** | 1.50 | 0.05 |
| **TA-01** | 3.48 | 1.10 |
| **TA-02** | 2.12 | 0.84 |
| **TA-03** | 1.57 | 0.32 |
| **TA-04** | 0.42 | 0.11 |
| **TA-05** | 4.48 | 1.76 |
| **TA-06** | 0.17 | 0.01 |
| **TA-07** | 0.83 | 0.09 |
| **TA-08** | 0.15 | 0.06 |
| **TA-09** | 0.13 | - |
| **TA-10** | 0.05 | - |
| **TA-11** | 0.04 | - |
| **TA-12** | 0.03 | - |
| **TA-13** | 0.05 | 0.01 |
| **TA-14** | 0.17 | 0.04 |
| **TA-15** | 2.43 | 0.70 |
| **TA-16** | 0.57 | 0.16 |
| **TA-17** | 1.23 | 0.30 |
| **TA-18** | 1.42 | 0.20 |
| **TA-19** | 0.54 | 0.04 |
| **TA-20** | 0.06 | 0.02 |
| **TA-21** | 2.38 | 0.05 |
| **TA-22** | 0.72 | 0.03 |
| **TA-23** | 0.72 | 0.06 |
| **TA-24** | 0.44 | 0.06 |
| **TA-25** | 2.13 | 0.47 |
| **TA-26** | 0.19 | 0.16 |
| **TA-27** | 0.32 | 0.02 |
| **TA-28** | 2.64 | 0.10 |
| **TA-29** | 0.15 | 0.03 |
| **TA-30** | 0.18 | 0.08 |
| **TA-31** | 8.64 | 0.18 |
| **TA-32** | 4.06 | 0.25 |
| **TA-33** | 0.55 | 0.12 |
| **TA-34** | 15.19 | 0.33 |
| **TA-35** | 2.64 | 0.55 |
| **TA-36** | 0.25 | 0.02 |
| **TA-37** | 1.44 | 0.03 |
| **TA-38** | 4.60 | 0.97 |
| **TA-39** | 1.04 | 0.18 |
| **TA-40** | 35.9 | 0.55 |
| **TA-41** | 5.77 | 0.20 |
| **TA-42** | 3.78 | 0.22 |
| **TA-43** | 0.74 | 0.04 |
| **TA-44** | 0.68 | 0.16 |
| **TA-45** | 2.71 | 0.14 |
| **TA-46** | 0.08 | - |
| **TA-48** | 1.62 | 0.49 |
| **TA-50** | 1.19 | 0.55 |
| **TA-51** | 1.16 | 0.54 |
| **TA-53** | 0.25 | 0.03 |
| **TA-54** | 2.17 | 0.57 |
| **TA-55** | 0.01 | - |
| **TA-58** | 9.16 | 0.24 |
