# Supplementary material for: Investigation of potential migratables from paper and board food contact materials
Source: Front Chem. 2023 Nov 30;11:1322811. doi: 10.3389/fchem.2023.1322811 (PMC10720245; doi:10.3389/fchem.2023.1322811)
Supplement: Supplementary file 5 [file Table2.docx]

**SUPPLEMENTARY DATA**

***Table S2: Target substances and detection parameters (retention times (RTs), collision energies (CE).***

| **Substance** | **CAS #** | **Abbreviation** | **Purity (%)** | **RT (min)** | **MS/MS Quantification transition** | | | **MS/MS Qualification transition** | | |
| --- | --- | --- | --- | --- | --- | --- | --- | --- | --- | --- |
|  |  |  |  |  | **Parent m/z** | **Product m/z** | **CE (eV)** | **Parent m/z** | **Product m/z** | **CE (eV)** |
| **Plasticizers** | | | | | | | | | | |
| Diisobutyl phthalate | 84-69-5 | DiBP | 99.0 | 11.6 | 149 | 65 | 25 | 149 | 93 | 15 |
| Dibutyl phthalate | 84-74-2 | DBP | 99.0 | 12.3 | 149 | 65 | 25 | 149 | 93 | 15 |
| Bis(2-ethylhexyl) phthalate | 117-81-7 | DEHP | 99.7 | 17.6 | 149 | 65 | 25 | 167 | 149 | 5 |
| Diisodecyl phthalate | 26761-40-0 | DiDP | 99.9 | 22.0 | 307 | 149 | 10 | 307 | 71 | 10 |
| Diisononyl phthalate | 28553-12-0 | DiNP | 99.0 | 22.0 | 293 | 149 | 10 | 293 | 71 | 10 |
| 1,2-Cyclohexane dicarboxylic acid diisononyl ester | 166412-78-8 | DINCH | 98.0 | 20.7 | 155 | 81 | 15 | 155 | 155 | 15 |
| Benzyl butyl phthalate | 85-68-7 | BBP | 98.4 | 16.7 | 149 | 65 | 25 | 149 | 121 | 10 |
| Di-iso-propyl phthalate | 605-45-8 | DiPrP | 98.7 | 10.1 | 149 | 65 | 25 | 149 | 93 | 15 |
| Bis-propyl ester phthalate | 605-45-8 | DPrP | 99.7 | 10.9 | 191 | 149 | 15 | 149 | 93 | 15 |
| Bis(4-methyl-2-pentyl) phthalate | 84-63-9 | BMPP | 99.7 | 13.6 | 149 | 65 | 25 | 149 | 93 | 15 |
| Bis(2-ethoxyethyl) phthalate | 605-54-9 | DEEP | 95.0 | 13.9 | 149 | 65 | 25 | 149 | 93 | 15 |
| Diisopentyl phthalate | 605-50-5 | DiPP | 99.3 | 12.4 | 149 | 65 | 25 | 149 | 93 | 15 |
| Dicyclohexyl phthalate | 84-61-7 | DCHP | 100 | 17.1 | 149 | 65 | 25 | 167 | 149 | 5 |
| Dipentyl phthalate | 131-18-0 | DPP | 99.0 | 14.3 | 149 | 65 | 25 | 149 | 93 | 15 |
| **Bisphenols** | | | | | | | | | | |
| Bisphenol A | 80-05-7 | BPA | 100 | 3.6 | 227 | 133 | 25 | 227 | 212 | 20 |
| Bisphenol S | 80-09-1 | BPS | 99.6 | 0.44 | 249 | 108 | 25 | 249 | 249 | 20 |
| Bisphenol Z | 843-55-0 | BPZ | 100 | 4.3 | 267 | 145 | 34 | 267 | 173 | 24 |
| Bisphenol B | 77-40-7 | BPB | 98.7 | 3.9 | 241 | 212 | 25 | 241 | 241 | 20 |
| Bisphenol F | 620-92-8 | BPF | 99.1 | 3.1 | 199 | 93 | 25 | 199 | 105 | 20 |
| **Primary aromatic amines** | | | | | | | | | | |
| Ortho-aminoazotoluene | 97-56-3 | o-AAT | 97.0 | 6.5 | 226 | 91 | 25 | 226 | 106 | 25 |
| 4-aminoazobenzene | 60-09-3 | 4-AAB | 96.9 | 5.8 | 198 | 77 | 30 | 198 | 93 | 30 |
| Aniline | 62-53-3 | ANL | 99.9 | 1.3 | 94 | 77 | 15 | 94 | 51 | 25 |
| o-anisidine | 90-04-0 | o-ASD | 99.0 | 2.7 | 124 | 109 | 15 | 124 | 65 | 25 |
| 4-chloro-aniline | 106-47-8 | 4-CA | 98.0 | 4.1 | 128 | 93 | 20 | 128 | 111 | 25 |
| 1,5-diaminonaphtalene | 2243-62-1 | 1,5-DAN | 100 | 1.9 | 159 | 143 | 25 | 159 | 115 | 40 |
| 3,3’-dichlorobenzidine | 91-94-1 | 3,3’-DCB | 99.3 | 5.7 | 254 | 218 | 20 | 254 | 183 | 30 |
| 2,2’-dichloro-4,4’ methylenedianiline | 101-14-4 | DCMDA | 90.7 | 5.8 | 268 | 232 | 20 | 268 | 141 | 40 |
| 2,4-dimethylaniline | 95-68-1 | 2,4-DMA | 99.3 | 3.3 | 122 | 107 | 15 | 122 | 105 | 15 |
| 2,6-dimethylaniline | 87-62-7 | 2,6-DMA | 99.2 | 4.3 | 122 | 105 | 15 | 122 | 77 | 20 |
| 3,3’-dimethylbenzidine | 119-93-7 | 3,3’-DMB | 99.1 | 3.6 | 213 | 198 | 20 | 213 | 181 | 30 |
| 3,3’-dimethoxybenzidine | 119-90-4 | 3,3’-DMOB | 97.6 | 3.5 | 245 | 187 | 30 | 245 | 230 | 30 |
| 4,4’-diaminodiphenylether | 101-80-4 | 4,4’-DPE | 99.3 | 1.9 | 201 | 108 | 20 | 201 | 80 | 25 |
| 4.4-diaminophenylmethane | 101-77-9 | 4,4’-MDA | 98.0 | 2.8 | 199 | 106 | 25 | 199 | 89 | 25 |
| 4,4’-methylene-di-o-toluidine | 838-88-0 | 4,4’-MDoT | 99.0 | 3.5 | 227 | 120 | 25 | 227 | 178 | 25 |
| 2-methyl-5-nitroaniline | 99-55-8 | 2-M-5-NA | 99.3 | 4.9 | 153 | 107 | 15 | 153 | 90 | 30 |
| 2,4-diaminoanisole | 615-05-4 | 2-M-mPDA | 100 | 0.88 | 139 | 108 | 15 | 139 | 124 | 15 |
| 2-methoxy-5-methylaniline | 120-71-8 | 2-MO-5-MA | 100 | 3.5 | 138 | 123 | 15 | 138 | 78 | 30 |
| m-phenylenediamine | 108-45-2 | m-PDA | 99.0 | 0.66 | 109 | 92 | 15 | 109 | 65 | 20 |
| p-phenylenediamine | 106-50-3 | p-PDA | 98.0 | 0.65 | 109 | 92 | 15 | 109 | 65 | 20 |
| 4,4’-thiodianiline | 139-65-1 | 4,4’-SDA | 99.6 | 4.0 | 217 | 124 | 20 | 217 | 200 | 20 |
| o-toluidine | 95-53-4 | o-T | 99.0 | 3.0 | 108 | 91 | 15 | 108 | 93 | 15 |
| 2,4-diaminotoluene | 95-80-7 | 2,4-TDA | 99.3 | 0.93 | 123 | 108 | 15 | 123 | 77 | 15 |
| 2,6-diaminotuoluene | 823-40-5 | 2,6-TDA | 98.0 | 0.85 | 123 | 106 | 15 | 123 | 79 | 15 |
| 2,4,5-trimethylaniline | 137-17-7 | 2,4,5-TMA | 99.4 | 3.7 | 136 | 121 | 20 | 136 | 91 | 20 |
| **Photoinitiators** | | | | | | | | | | |
| Benzophenone | 119-61-9 | BP | 99.9 | 3.3 | 183 | 105 | 15 | 183 | 77 | 30 |
| 1-chloro-4-propoxy-9H thioxanthen-9-one | 142770-42-1 | CPTX | 97.0 | 5.4 | 306 | 264 | 20 | 306 | 172 | 40 |
| 2-chloro-9H-thioxanthen-9-one | 86-39-5 | CTX | 98.0 | 4.8 | 248 | 213 | 20 | 248 | 185 | 25 |
| 4,4'-bis(diethylamino)benzophenone | 90-93-7 | DEAB | 99.0 | 4.6 | 325 | 176 | 25 | 325 | 281 | 25 |
| 2,4-diethyl-9H-thioxanthen-9-one | 82799-44-8 | DETX | 98.0 | 6.1 | 269 | 213 | 25 | 269 | 241 | 15 |
| 4-(dimethylamino)benzophenone | 530-44-9 | DMBP | 98.0 | 3.3 | 227 | 212 | 15 | 227 | 135 | 30 |
| 2,2-dimethoxy-2-phenyl acetophenone | 24650-42-8 | DMPA | 99.0 | 3.5 | 225 | 197 | 10 | 225 | 165 | 20 |
| 2-ethylanthraquinone | 84-51-5 | EA | 97.0 | 4.5 | 237 | 209 | 25 | 237 | 152 | 30 |
| 2-Ethylhexyl-4-dimethylaminobenzoate | 21245-02-3 | EDB | 98.0 | 6.4 | 278 | 134 | 25 | 278 | 107 | 20 |
| Ethyl-4-dimethylaminobenzoate | 10287-53-3 | EDMAB | 99.0 | 3.1 | 194 | 134 | 30 | 194 | 151 | 20 |
| 4-Hydroxybenzophenone | 1137-42-4 | 4-HBP | 99.9 | 1.9 | 199 | 121 | 20 | 199 | 93 | 35 |
| 1-hydroxylcyclohexyl phenylketone | 947-19-3 | HCPK | 99.7 | 2.9 | 187 | 105 | 15 | 187 | 77 | 20 |
| 2-Hydroxy-4methoxybenzophenone | 131-57-7 | HMBP | 99.8 |  | 229 | 151 | 15 | 229 | 105 | 20 |
| 2-hydroxy-4'-(2-hydroxyethoxy)-2-methylpropiophenone | 106797-53-9 | HMMP | 98.1 | 0.81 | 225 | 179 | 10 | 225 | 107 | 30 |
| 2-isopropyl-9H-thioxanthen-9-one 4-isopropyl-9H-thioxanthen-9-one | 5495-84-1  83846-86-0 | 2-ITX  4-ITX | 99.5 | 5.5 | 255 | 213 | 15 | 255 | 184 | 30 |
|  |  |  | 99.6 |  |  |  |  |  |  |  |
| 2-methylbenzophenone;  3-methylbenzophenone;  4-methylbenzophenone | 131-58-8  643-65-2  134-84-9 | 2-MBP  3-MBP  4-MBP | 99.0 | 3.8 | 197 | 105 | 10 | 197 | 77 | 30 |
|  |  |  | 98.9 |  |  |  |  |  |  |  |
|  |  |  | 98.5 |  |  |  |  |  |  |  |
| Michler's ketone | 90-94-8 | MK | 98.0 | 3.4 | 269 | 254 | 25 | 269 | 148 | 15 |
