# Supplementary material for: Investigation of potential migratables from paper and board food contact materials
Source: Front Chem. 2023 Nov 30;11:1322811. doi: 10.3389/fchem.2023.1322811 (PMC10720245; doi:10.3389/fchem.2023.1322811)
Supplement: Supplementary file 6 [file Table5.docx]

**SUPPLEMENTARY DATA**

***Table S5: Gas chromatographic and mass spectrometer parameters***

| **CHROMATOGRAPHIC PARAMETERS** | |
| --- | --- |
| **GC system** | Agilent 8890 |
| **Volume of injection** | 1 µL |
| **Injection mode** | Splitless |
| **Column** | HP-5 ms (0.25 µm ; 0.25 mm x 30 m) |
| **Oven temperature program** | 60°C hold 1.5 min  At 20°C/min to 220°C, hold 1 min  At 5°C/min to 280°C, hold 4 min |
| **Carrier gas** | Helium |
| **Flow rate** | 1.20 mL/min |
| **MS/MS PARAMETERS** | |
| **Mass system** | Agilent 7010B |
| **Solvent delay** | 5.0 min |
| **Ion source** | EI |
| **Ionization voltage** | 70 eV |
| **Collision gas** | Nitrogen 1.0 mL/min |
| **Quenching gas** | Helium 1.00 mL/min |
