# Supplementary material for: Investigation of potential migratables from paper and board food contact materials
Source: Front Chem. 2023 Nov 30;11:1322811. doi: 10.3389/fchem.2023.1322811 (PMC10720245; doi:10.3389/fchem.2023.1322811)
Supplement: Supplementary file 7 [file Table3.docx]

**SUPPLEMENTARY DATA**

***Table S3: Example of chromatograms***

*Photoinitiators (Calibration 4)*


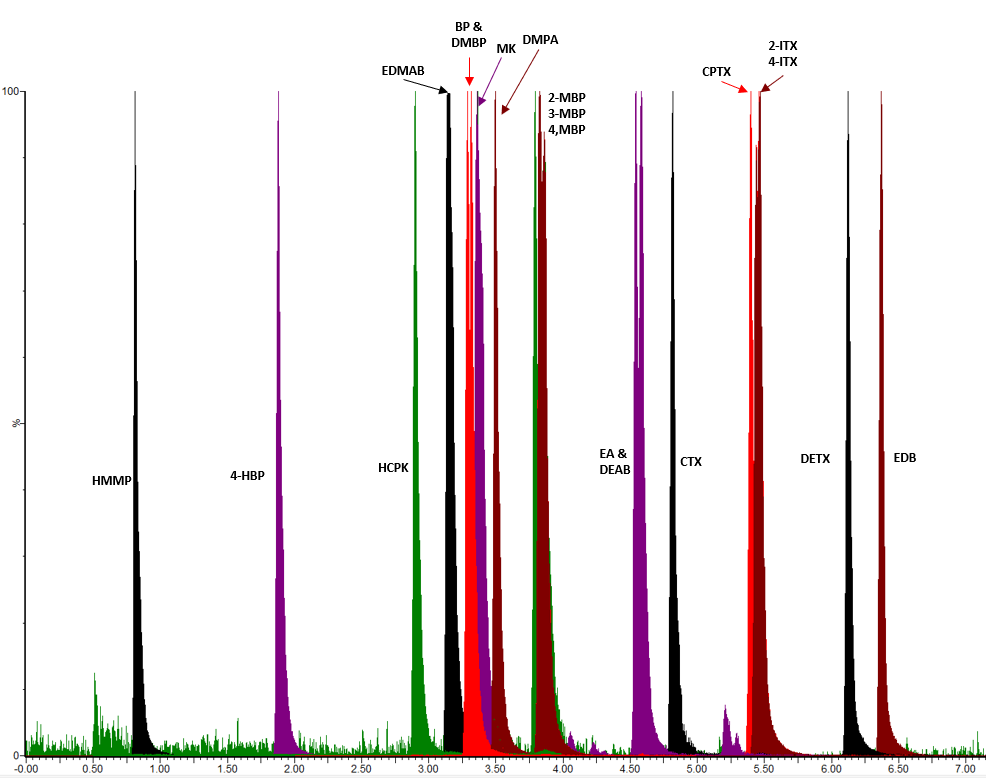


*Bisphenols (Calibration 4)*


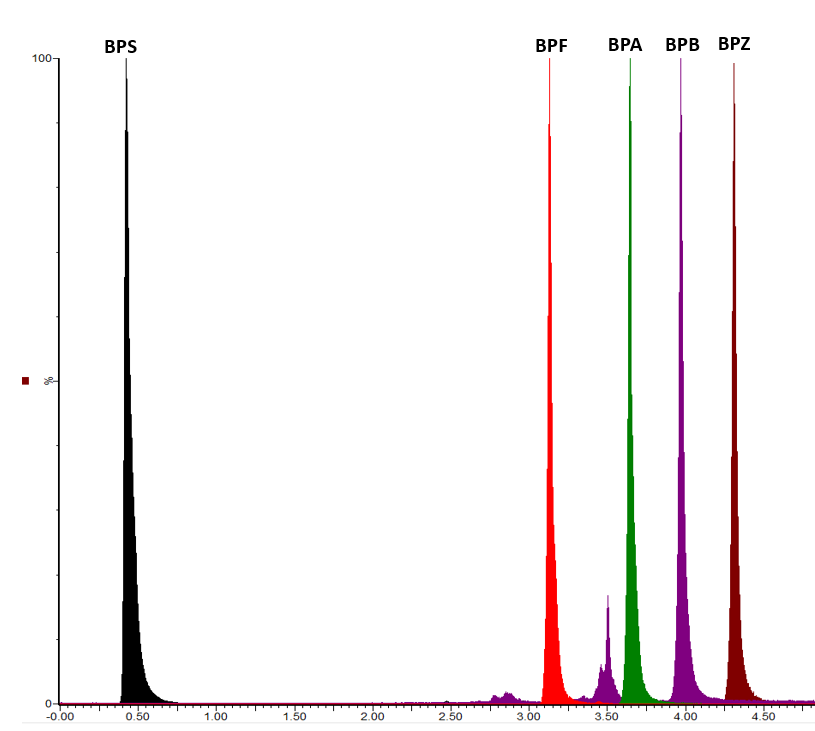


*Primary aromatic amines (Calibration 7)*


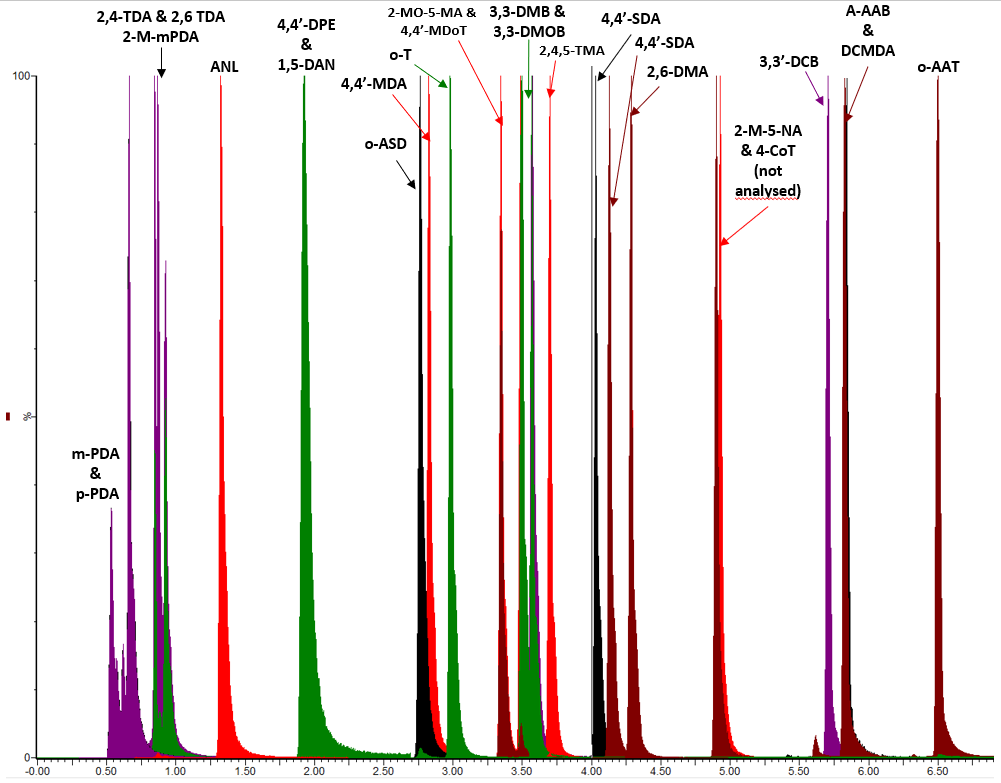


*Plasticizers (Cal 7)*


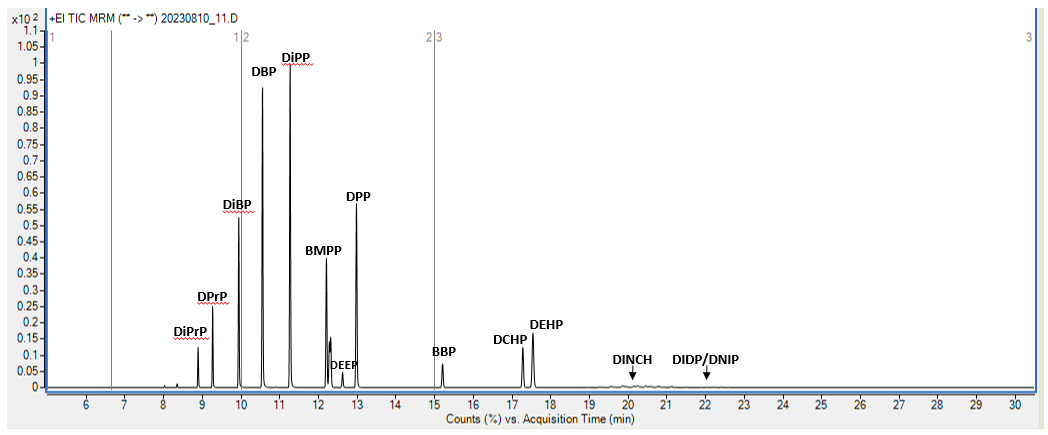


*MOSH/MOAH (Sample TA-05)*

***
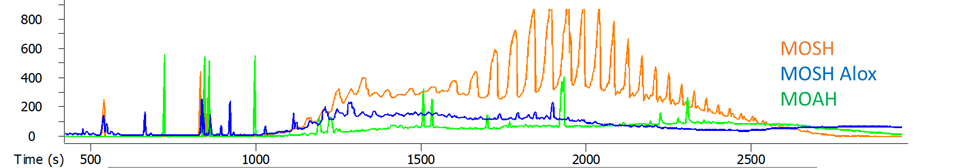
***
