# Supplementary material for: Investigation of potential migratables from paper and board food contact materials
Source: Front Chem. 2023 Nov 30;11:1322811. doi: 10.3389/fchem.2023.1322811 (PMC10720245; doi:10.3389/fchem.2023.1322811)
Supplement: Supplementary file 8 [file Table4.docx]

**SUPPLEMENTARY DATA**

***Table S4: Liquid chromatographic and mass spectrometer parameters***

| **Instrument** | Acquity™ UPLC H-Class (Waters) | | | | | | | |
| --- | --- | --- | --- | --- | --- | --- | --- | --- |
| **Column** | Bisphenols: InfinityLab Poroshell 120 EC-C18 (1.9µM;2.1x100mm)  PAA + PIs : Acquity UPLC BEH C18(1.7 µM; 2.1x 100 mm) | | | | | | | |
| **Volume of injection** | Bisphenols : 5 µL  PAA: 10 µL  PIS : 10 µL | | | | | | | |
| **Flow rate** | 0.4 mL/min | | | | | | | |
| **Column temperature** | Bisphenols: 45°C | | PAA: 40°C | | | | PIs: 27.5°C | |
| **Autosampler temperature** | 10°C | | | | | | | |
| **Mobile phases** | Bisphenols  A : 0.1% NH3 in H20  B : 0.1% NH3 in ACN | PAA  A: Milli-Q Water  B: 0.1% FA in ACN | | | | PIs  A: 0.1 FA in Milli-Q Water  B: 0.1 FA in ACN | | |
| **Gradient PIs (ESI +)** | **Time (min)** | | | **%A** | **%B** | | | **Curve** |
|  | 0 | | | 60 | 40 | | | 1 |
|  | 6 | | | 0 | 100 | | | 6 |
|  | 8 | | | 0 | 100 | | | 6 |
|  | 10 | | | 60 | 40 | | | 1 |
| **Gradient PAA (ESI +)** | **Time (min)** | | | **%A** | **%B** | | | **Curve** |
|  | 0 | | | 95 | 5 | | | Initial |
|  | 1 | | | 95 | 5 | | | 6 |
|  | 6 | | | 5 | 95 | | | 6 |
|  | 8 | | | 5 | 95 | | | 6 |
|  | 10 | | | 95 | 95 | | | 1 |
| **Gradient Bisphenols (ESI -)** | **Time (min)** | | | **%A** | **%B** | | | **Curve** |
|  | 0 | | | 95 | 5 | | | 6 |
|  | 0.5 | | | 95 | 5 | | | 6 |
|  | 2.5 | | | 50 | 50 | | | 6 |
|  | 5 | | | 5 | 95 | | | 6 |
|  | 5.5 | | | 5 | 95 | | | 6 |
|  | 6 | | | 95 | 5 | | | 6 |
|  | 6.5 | | | 95 | 5 | | | 6 |
