# Supplementary material for: Investigation of potential migratables from paper and board food contact materials
Source: Front Chem. 2023 Nov 30;11:1322811. doi: 10.3389/fchem.2023.1322811 (PMC10720245; doi:10.3389/fchem.2023.1322811)
Supplement: Supplementary file 9 [file Table6.docx]

**SUPPLEMENTARY DATA**

***Table S6 : Hypothesis of use/consumption of food***

| **Description** | **Hypothesis - frequency of use children** | **Hypothesis - frequency of use teenagers** | **Hypothesis - frequency of use adults** |
| --- | --- | --- | --- |
| Straws | 5x per week | 1x per week | 1x per month |
| Pizza box | 1x per week | 2x per week | 1x per week |
| Noodle box | 1x per month | 1x per week | 2x per week |
| Fries trays/bag | 2x per week | 2x per week | 2x per week |
| Hamburger wrap | 1x per week | 1x per week | 1x per week |
| Paper snack and sandwich bag | 1x per month | 5x per week | 5x per week |
| Snack tray | 1x per week | 1x per week | 1x per week |
| Paper spoon | 1x per week | 2x per week | 1x per week |
| Takeaway Cardboard Box | 1x every 2 weeks | 5x per week | 5x per week |
| Hamburger box | 1x per week | 1x per week | 1x per week |
| Soup bowl | 3x per week | 5x per week | 5x per week |
| Ice cream bowl | 1x per week | 1x per week | 1x per week |
| Bowl | 1x every 2 weeks | 5x per week | 5x per week |
| Coffee cup/cup | 1x per week | 10x per week | 10x per week |
